# An Analysis of Trafficking Receptors Shows that CD44 and P-Selectin Glycoprotein Ligand-1 Collectively Control the Migration of Activated Human T-Cells

**DOI:** 10.3389/fimmu.2017.00492

**Published:** 2017-05-03

**Authors:** Amal J. Ali, Ayman F. Abuelela, Jasmeen S. Merzaban

**Affiliations:** ^1^King Abdullah University of Science and Technology (KAUST), Division of Biological and Environmental Sciences and Engineering (BESE), Thuwal, Saudi Arabia

**Keywords:** E-selectin, CD44, hematopoietic cell E- and/or L-selectin ligand, P-selectin glycoprotein ligand-1 (CD162), cell adhesion, cell migration, human activated T-cells, psoriasis

## Abstract

Selectins guide the traffic of activated T-cells through the blood stream by mediating their tethering and rolling onto inflamed endothelium, in this way acting as beacons to help navigate them to sites of inflammation. Here, we present a comprehensive analysis of E-selectin ligands expressed on activated human T-cells. We identified several novel glycoproteins that function as E-selectin ligands. Specifically, we compared the role of P-selectin glycoprotein ligand-1 (PSGL-1) and CD43, known E-selectin ligands, to CD44, a ligand that has not previously been characterized as an E-selectin ligand on activated human T-cells. We showed that CD44 acts as a functional E-selectin ligand when expressed on both CD4^+^ and CD8^+^ T-cells. Moreover, the CD44 protein carries a binding epitope identifying it as hematopoietic cell E- and/or L-selectin ligand (HCELL). Furthermore, by knocking down these ligands individually or together in primary activated human T-cells, we demonstrated that CD44/HCELL, and not CD43, cooperates with PSGL-1 as a major E-selectin ligand. Additionally, we demonstrated the relevance of our findings to chronic autoimmune disease, by showing that CD44/HCELL and PSGL-1, but not CD43, from T-cells isolated from psoriasis patients, bind E-selectin.

## Introduction

Recruitment and infiltration of T-cells are necessary for the development and maintenance of the immune response. Immune dysregulation occurs when the natural response to protect against pathogens and injuries is disrupted, a state that can lead to autoimmunity. Psoriasis, characterized by painful red skin lesions, is an autoimmune skin disease that affects about 2–5% of the world population (1, 2). Activated T-cells are key players in mediating the immune response causing skin diseases, such as psoriasis (3, 4), likely explained by their tendency to upregulate functional selectin ligands and chemokines that are associated with their migration to skin (5, 6). This migration requires that activated T-cells tether and roll onto selectins expressed on the endothelium ultimately leading to transendothelial migration following a period of arrest (6–8). Tethering and rolling are highly dependent on the ability of activated T-cells to express glycoproteins that reversibly and transiently bind to P- and E-selectins (9–11). In mice, both selectins have overlapping and partially redundant functions (12, 13). In human, however, P-selectin is only minimally expressed at sites of leukocyte infiltration, such as at dermal venules, and where hematopoietic stem/progenitor cells (HSPCs) infiltrate at bone marrow sinusoids (14, 15). Moreover, the ability of some inflammatory mediators, such as tumor necrosis factor and lipopolysaccharides, to induce the expression of human E-selectin, but not P-selectin, limits the relevance of P-selectin-mediated skin inflammatory responses in humans (11, 15–17).

To date, only two E-selectin ligands have been reported on activated human T-cells: P-selectin glycoprotein ligand-1 (PSGL-1; CD162) (18) and CD43 (19, 20). In mice, *in vivo* studies have illustrated that a concomitant deficiency of these ligands is not sufficient to completely eliminate E-selectin-dependent migration of activated T-cells, suggesting other ligands are present (20, 21). In this study, we utilized the power of mass spectrometry to identify unknown E-selectin ligands expressed on the surface of activated human T-cells. Using this technology, we detected a repertoire of glycoproteins that bind to recombinant E-selectin protein. In addition to the previously described ligands, PSGL-1 and CD43, we also identified CD44 on activated human T-cells. CD44 is a structurally variable cell surface glycoprotein that ranges in size from 85 to 250 kDa. This variability is mediated by alternative splicing as well as extensive posttranslational modifications including *N*- and *O*-glycosylation and glycosaminoglycan attachment (22). The most abundant form of CD44 is the standard form (CD44s; 85–95 kDa), which lacks the variable exons (v2–v10 and exon 18) (23), and it is mostly *N*-glycosylated (24). CD44s is also designated as CD44H because it is expressed mainly on cells of lympho-hematopoietic origin (25). CD44 marks activated and memory T-cells and is reported to function as an E-selectin ligand on HSPCs, neutrophils, and mouse T helper-1 cells (26–29). Furthermore, binding of CD44 to hyaluronic acid (HA) increases both T-cell proliferation and extravasation into inflammatory sites (30, 31); however, its role as an E-selectin ligand on human T-cells has not previously been established. In fact, it is reported that CD44 from CLA^+^ human T-cells does not interact with E-selectin (19). Although CD44 was reported as an E-selectin ligand on mouse T helper-1 cells, extrapolating studies from mouse to humans should be done with caution due to the significant differences between the human and mouse immune systems (29, 32, 33). Therefore, we found it crucial to explore the role of CD44 as a functional E-selectin ligand on human activated T-cells. Here, we demonstrated that CD44 isolated from human activated CD4^+^ and CD8^+^ T-cells is decorated with sialofucosylated glycans and binds to E-selectin, which thereby defines it as the specialized glycoform, hematopoietic cell E- and/or L-selectin ligand (HCELL) (26, 34). Moreover, *in vivo*-activated T-cells isolated from psoriasis patients strongly suggest that both PSGL-1 and CD44, but not CD43, are the major E-selectin ligands. Using a small interfering RNA (siRNA) knockdown approach in primary activated human T-cells, we demonstrated that CD44 cooperates with PSGL-1 to maintain and support T-cell rolling under physiological flow conditions. This work has significant implications in the development of targeted therapies to combat inflammatory diseases where T-cell trafficking is more dependent on E-selectin expression (i.e., psoriasis, rheumatoid arthritis) to selectively interfere with the pathophysiology of these diseases through the targeting of a subset of these major E-selectin ligands.

## Results

### CD44 Is an E-Selectin Ligand on Human Activated T-Cells

**Figure 1:**
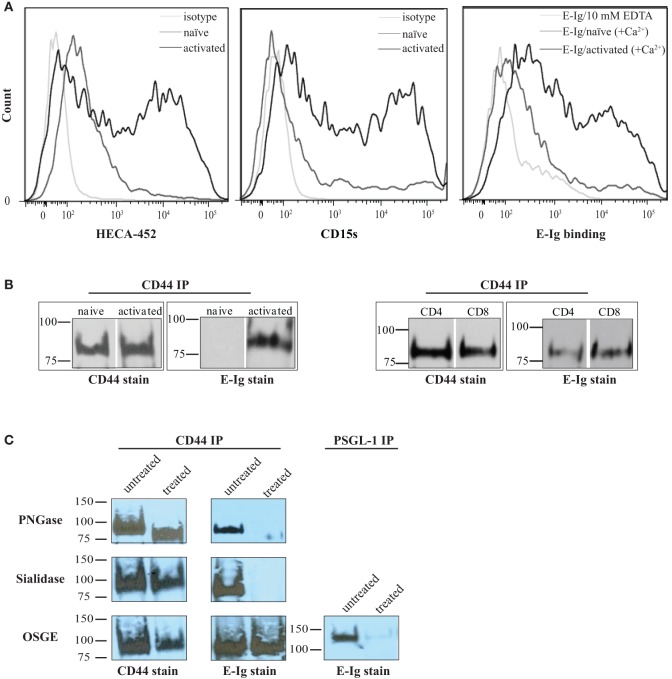
**CD44/HCELL isolated from activated human T-cells serves as an E-selectin ligand**. **(A)** sLe^x^ expression was assayed by the reactivity of activated (black) and naïve (dark-gray) T-cells to HECA-452 (*left panel*) or CD15s (*middle panel*) monoclonal antibodies. The isotype controls were included as negative controls (light-gray). E-Ig binding to activated (black) versus naïve (dark-gray) human T-cells (r*ight panel*) is shown in the *right panel*. To assay for non-specific interactions, 10-mM EDTA (light-gray) was added to the assay buffer. These are representative flow cytometric histograms of *n* = 3 independent experiments. **(B)** CD44 was immuno-purified from naïve and activated T-cells (*right panel*) and from activated human CD4^+^ and CD8^+^ T-cells (*left panel*) purified using autoMACS (Figure S1 in Supplementary Material). The samples were prepared for Western blot analysis and stained with either CD44 or E-Ig (or secondary alone controls; Figure S4 in Supplementary Material). E-Ig specifically binds to CD44 immuno-purified from both activated CD4^+^ and CD8^+^ T-cells but not naïve T-cells. These are representative blots of *n* = 3 independent experiments. **(C)** CD44 was immuno-purified from activated T-cells that were either untreated or treated with PNGaseF to remove *N*-glycans (*upper panels*), sialidase to remove sialic acid (*middle panels*), or OSGE to remove *O*-glycans (*lower panels*) and prepared for Western blot analysis for CD44 expression or for binding to E-Ig. Note that the data suggest that the interaction between E-selectin and CD44 is *N*-glycan and sialic acid dependent but that it is not affected by the removal of *O*-glycans. The efficiency of OSGE enzymatic treatment was confirmed by its ability to abolish the E-selectin binding capacity of P-selectin glycoprotein ligand-1 (PSGL-1) after the removal of *O*-glycan. These are representative blots of *n* = 3 independent experiments.

Activation of naïve human T-cells results in the expression of selectin ligands (35, 36). Selectins bind to specialized sialofucosylated carbohydrate determinants, prototypically displayed as the terminal tetrasaccharide sialyl Lewis structures (sLe^x^; and/or to its isomer sLe^a^) (34, 37). Flow cytometric analysis of sialyl Lewis x/a expression on human activated T-cells was confirmed using specific monoclonal antibodies (mAbs), HECA-452 and CD15s (18, 27), that were shown to markedly increase after activation subsequently leading to E-selectin binding [E-selectin-hIg chimera (E-Ig); Figure 1A]. Overall, these results suggest that human T-cells were appropriately stimulated in culture to express functional E-selectin ligands.

In order to fully elucidate the potential repertoire of E-selectin ligands on the surface of activated human T-cells, we immuno-purified the ligands using E-Ig and the products were analyzed by mass spectrometry; of the 32 proteins detected (Table S1 in Supplementary Material), 10 were identified as cell membrane glycoproteins that could bind E-selectin (Table 1). The role of CD44 in many cellular processes, including growth, survival, differentiation, and migration of hematopoietic cells (18, 27, 34, 38, 39), motivated us to substantiate its involvement in E-selectin-mediated T-cell migration. To this end, we immuno-purified equal amounts of CD44 from naïve and activated T-cells (Figure 1B) and assessed E-Ig-binding activity by Western blot. As illustrated in Figure 1B (*left*), the CD44 isolated from naïve T cells is non-functional and gained E-selectin binding activity only upon T-cell activation. To identify the T-cell subset that expressed E-selectin binding activity toward CD44, we purified CD4 and CD8 cells from the activated T-cell pool with more than 90% purity (Figure S1 in Supplementary Material) and subsequently immuno-purified CD44 from each population; CD44 from both subsets showed E-selectin binding (Figure 1B, *right*). Moreover, T-cell phenotyping of the CD4^+^ subset identified them as mainly of Th2 phenotype (Table S2 in Supplementary Material). HCELL is recognized as a sialofucosylated glycoform of CD44, originally found on HSPCs, that acts as an E-selectin and/or L-selectin ligand (26, 34). Our analysis shows that CD44 from activated human T-cells bind exclusively to E-selectin and not to L- or P-selectin (Figure S2 in Supplementary Material). In the work outlined here, CD44 will be considered as HCELL (and designated CD44/HCELL) since it stains positive for sialofucosylated glycans, albeit weaker than PSGL-1 (Figure S3 in Supplementary Material), and binds E-selectin.

**Table 1:** **E-selectin ligands were immuno-purified from activated human T-cell lysate and the purified proteins were subjected to mass spectrometry analysis for ligand identification**.

Protein name	Peptide sequence
Leukosialin (CD43)	NGVVDAWAGPAQVPEEGAVTVTVGGSGGDKGSGFPDGEGSSR/QGSLAMEELK/TGALVLSR
P-selectin glycoprotein ligand-1	SPGLTPEPR
CD44 antigen	ESSETPDQFMTADETR/LVINSGNGAVEDR/NLQNVDMK
T-cell differentiation antigen CD6	HRVTDEEVQQSR/VTDEEVQQSR
Facilitated glucose transporter member 1	GTADVTHDLQEMKEESR/QGGASQSDKTPEELFHPLGADSQV/TFDEIASGFR
Dipeptidyl peptidase 4	LGTFEVEDQIEAAR/VLEDNSALDK
Facilitated glucose transporter member 3	AFEGQAHGADR/LWGTQDVSQDIQEMKDESAR
Transferrin receptor protein 1	LAVDEEENADNNTK/LLNENSYVPR/SSGLPNIPVQTISR
4F2 cell surface antigen heavy chain	ADLLLSTQPGREEGSPLELER/IKVAEDEAEAAAAAK/VAEDEAEAAAAAK
HLA class I histocompatibility antigen, Cw-12 alpha chain	DGEDQTQDTELVETRPAGDGTFQK/FDSDAASPR/WAAVVVPSGEEQR/FIAVGYVDDTQFVR

*Lists all the identified membrane glycoproteins along with their corresponding identified peptide sequence(s)*.

To characterize the contribution of glycans (38) to E-selectin binding on CD44/HCELL, immuno-purified CD44 was treated with enzymes to remove *N*- or *O*-glycans (PNGaseF or OSGE, respectively) prior to Western blot analysis for E-selectin binding (with E-Ig). As evident in Figure 1C, the E-selectin interaction with CD44/HCELL was abolished when *N*-glycans were removed (Figure 1C, *top*) but not when *O*-glycans were removed (Figure 1C, *bottom*). CD44/HCELL treated with PNGaseF displayed altered mobility on SDS-PAGE confirming the efficacy of the treatment (Figure 1C). As CD44/HCELL is not highly *O*-glycosylated, we did not detect a change in mobility following OSGE treatment but to confirm the efficacy of removal of *O*-glycans, we illustrated that PSGL-1 binding to E-selectin was abolished (Figure 1C) since this interaction is *O*-glycan dependent. Finally, treatment with sialidase to remove the sialic acid completely abolished the interaction of E-selectin with CD44/HCELL (Figure 1C, *middle*). These experiments demonstrated that E-selectin recognizes *N*-linked sialylated glycans on CD44/HCELL of activated human T-cells much similar to that of HSPCs (26, 27, 34, 40).

**Figure 2:**
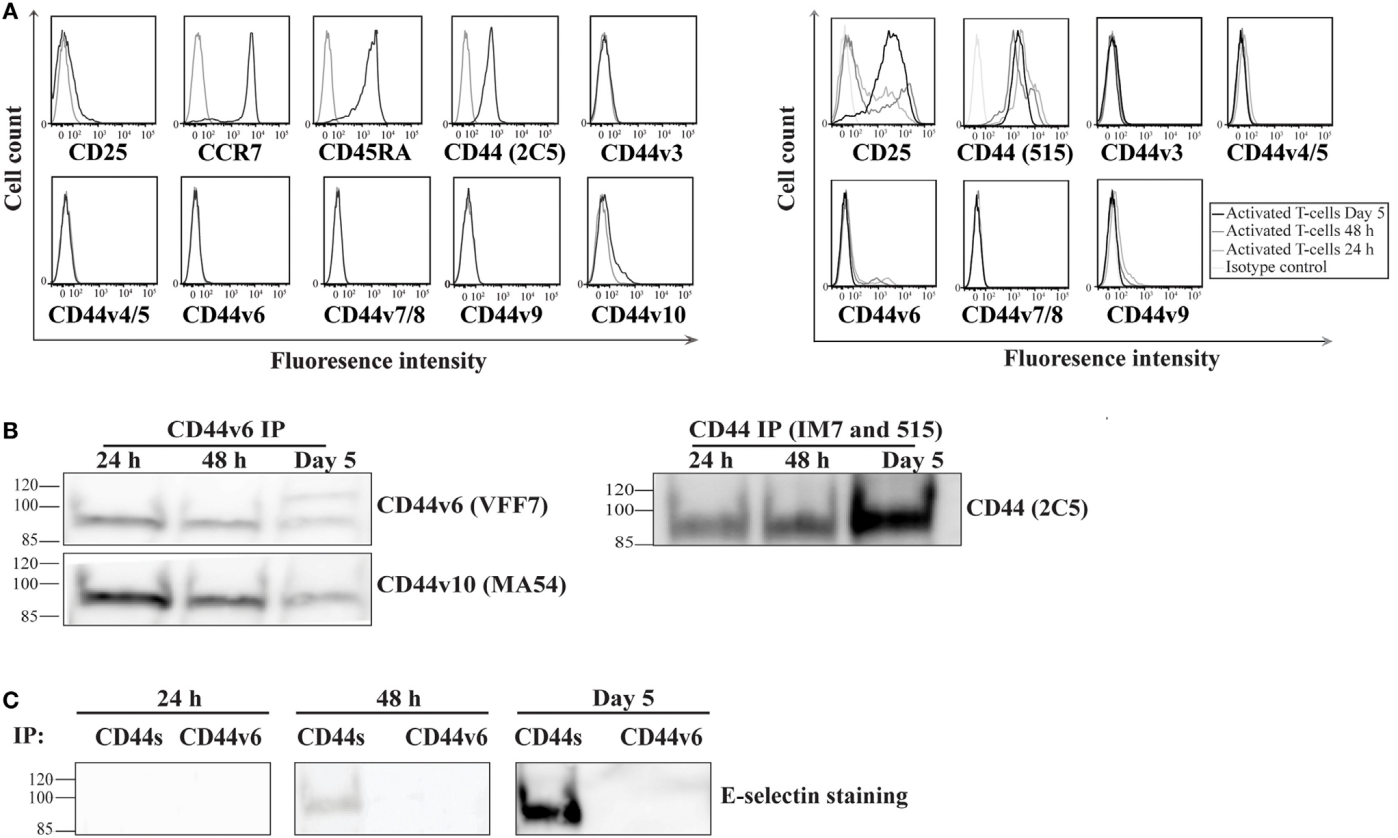
**CD44s, not CD44 variant (CD44v), is an E-selectin ligand on activated human T-cells**. **(A)** To analyze the expression of CD44v on the surface of naive (*left panel*; CD25^−^CCR7^+^CD45RA^+^) and activated (*right panel*) T-cells at different time points after activation (24 h, 48 h, and Day 5), the cells were incubated with monoclonal antibodies against specific CD44v isoforms (black line) or isotype controls (gray line) and analyzed by flow cytometry. The activity of the CD44v antibodies were confirmed using breast cancer cell lines (Figure S5 in Supplementary Material). **(B)** CD44v6 (*left panel*) and CD44s (*right panel*) were consecutively immuo-purified from cell lysates prepared at different time points (24 h, 48 h, and Day 5), subjected to Western blot analysis and stained with CD44v6 and CD44v10 (*left panel*) and CD44s (*right panel*). The protein immuno-purified by the anti-CD44v6 also stained positive for CD44v10 (*left panel*), which indicates that activated T-cells express a form of CD44 that comprises both exon 6 and exon 10. **(C)** Western blot analysis of immuno-purified CD44s and CD44v6 illustrates that E-selectin interacts with CD44s but not CD44v6/10. This interaction was time dependent initially detected at 48 h and increasing significantly at Day 5. Immuno-purification for CD44 (s and v) was performed from cell lysates that were normalized for cell number at each time point (24 h, 48 h, and Day 5). Data are representative of *n* = 3 independent experiments.

Recent studies have underscored the importance of the CD44 variant (CD44v) isoforms in mediating E-selectin binding of a number of cancer cell lines (24, 41). Therefore, we sought to investigate whether CD44s and/or CD44v are involved in E-selectin-binding activity on activated human T-cells. To determine which isoform is expressed on activated human T-cells, we initially screened naïve and activated T-cells for CD44 expression using either anti-CD44 mAbs that recognize both the standard and variant CD44 isoforms (clones 515 and 2C5) or that are specific for each of the variant isoforms alone. Our flow cytometric analysis illustrated that the majority of CD44 expressed on naïve T-cells is of the CD44s isoform and a small amount is of the CD44v10 isoform (Figure 2A, *left panel*). Following activation, the majority of CD44 remains of the CD44s isoform with a small population of cells that express the CD44v6 isoform (i.e., no CD44v3–5/7–9 is detected) (Figure 2A, *right panel*). A time course analysis of activated cells showed that the expression of the CD44v6 peaked at 24–48 h after activation and decreased at Day 5. To determine whether CD44v6 or CD44s possessed E-selectin ligand activity, we serially immuno-purified CD44v6 (Figure 2B, *left panel*), CD44v10, and finally CD44s (Figure 2B, *right panel*) from the same lysate at different time points after activation (24 h, 48 h, and Day 5), and then we conducted a Western blot analysis. Figure 2B shows that the anti-CD44v6 mAb immuno-purified a protein that expresses exon 6 (stained with CD44v6 mAb) and exon 10 (stained with CD44v10 mAb) from activated T-cell lysates, which implies that the transcript(s) that express CD44v6 also harbor CD44v10 (Figure 2B, *left panel*). Interestingly, the expression of CD44s increases with time from 24 h to Day 5 of the culture (Figure 2B, *right panel*). E-selectin binding studies show that CD44s, but not CD44v6/10, bind to E-selectin and that the binding was time dependent starting at 48 h increasing dramatically by Day 5 of the culture (Figure 2C).

**Figure 3:**
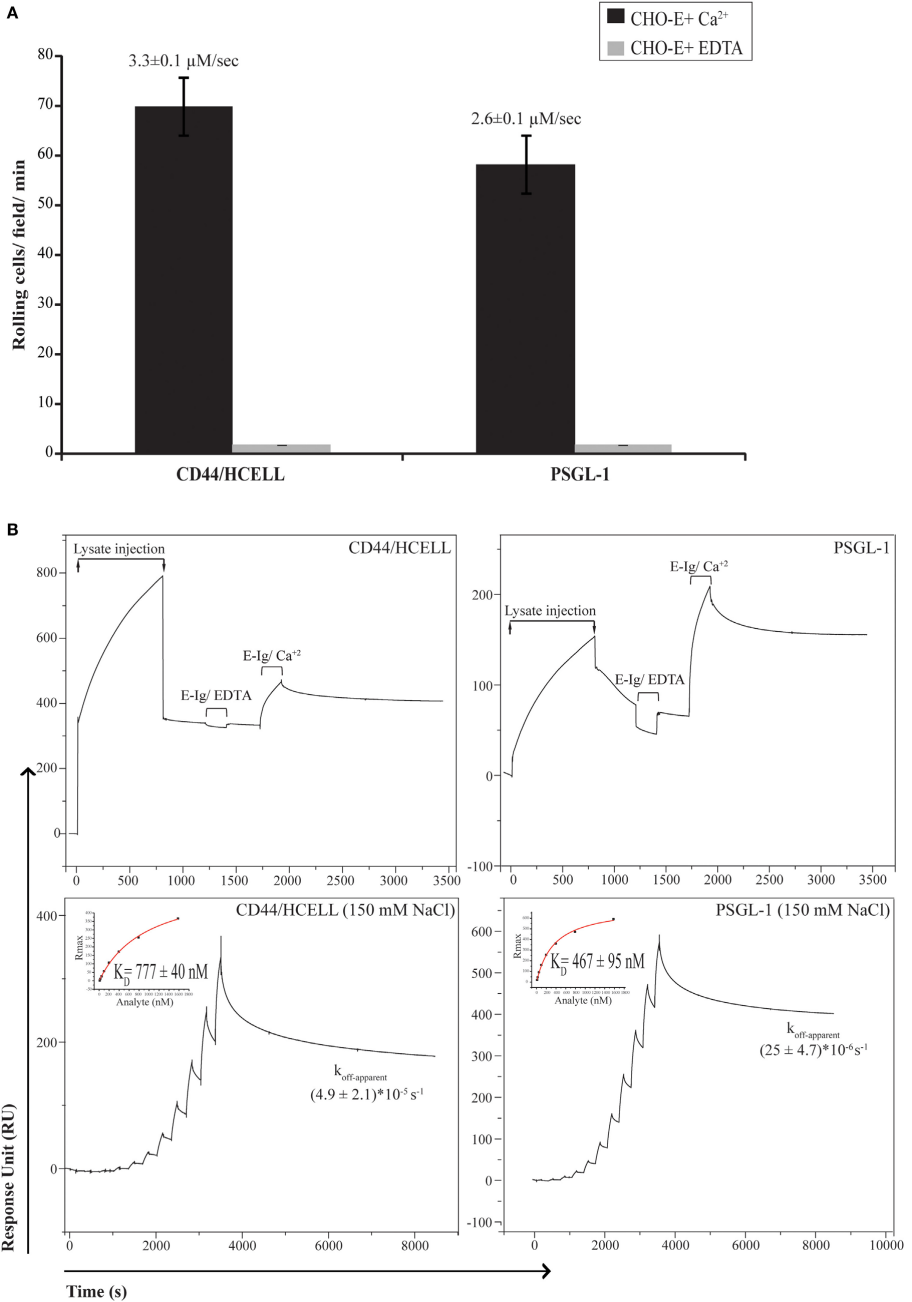
**CD44/HCELL is a functional E-selectin ligand on activated human T-cells**. **(A)** Blot rolling assays were performed on immuno-purified CD44/HCELL and P-selectin glycoprotein ligand-1 (PSGL-1). Initially, immuno-purified protein was resolved by SDS-PAGE. Then, CHO-E cells were allowed to roll over immuno-purified proteins in the presence of 2-mM Ca^2+^ at 0.5 dyn cm^−2^. After cell perfusion, the numbers of rolling cells per square millimeter in five distinct fields of view were counted and the average is presented (*black bars*). The specificity of CHO-E binding to membrane glycoproteins was assessed by adding 5-mM EDTA to the buffer containing CHO-E cells (*gray bars*). The *x*-axis reflects the mean of rolling cells per field per minute from one representative experiment of multiple independent membrane preparations (*n* = 3). The mean velocity ± SEM of rolling cells was determined and presented here on top of the bars. **(B)** BIAcore assay was performed to determine the ability of native CD44/HCELL and PSGL-1 to bind E-Ig. To determine the ability of captured protein to bind E-Ig, we injected 100-nM of E-Ig at 20 μl min^−1^ over the native CD44/HCELL or PSGL-1 (*upper panels*) in the presence of 5-mM EDTA to control for specificity or 1-mM Ca^2+^ to study the binding. To determine the kinetics of the CD44/HCELL interaction with E-Ig and compare it to that with PSGL-1 (*lower panels*), monoclonal antibodies or their isotype controls were immobilized on the chip, and then the ligands were captured. Afterward, a series of 10 E-Ig concentrations (0.456–466.7 nM) were injected at 30 μl min^−1^ over CD44/HCELL or PSGL-1 in 150-mM NaCl running buffer. The equilibrium dissociation constant (*K*_D_) was derived from fitting the binding isotherm using the steady-state model and the maximum response unit (RUmax) of E-Ig binding just before the washing step. After the last E-Ig injection, we determined the apparent dissociation rate constant (*K*_off-apparent_) by fitting the stable phase obtained during the buffer wash. Data shown here are the mean ± SEM of *n* = 3, after the correction against the bulk refractive index and subtraction of the non-specific binding of the isotype control.

To determine if CD44/HCELL supports E-selectin binding under physiological flow, we used the blot rolling assay, which allowed us to compare the ability of immuno-purified CD44/HCELL and PSGL-1 (positive control) isolated from the same activated T-cell lysate to support the rolling of CHO-E cells under defined shear flow conditions. As illustrated in Figure 3A (Videos S1 and S2 in Supplementary Material), CHO-E cells specifically interacted with CD44/HCELL and PSGL-1 and this binding was Ca^2+^ dependent as it was lost when cells were suspended in 5-mM EDTA buffer. The average velocities of the cells rolling over CD44/HCELL and PSGL-1 in five fields/ligands were comparable (3.3 ± 0.1 μM s^−1^ for CD44 and 2.6 ± 0.1 μM s^−1^ for PSGL-1, Figure 3A). Thus, CD44/HCELL displayed Ca^2+^-dependent E-selectin ligand activity under flow conditions comparable to PSGL-1.

Next, we used a recently developed novel and robust real-time immunoprecipitation assay (40) to perform a comparative analysis for measuring the interaction of native ligands expressed on human activated T-cells with E-Ig. Both CD44 and PSGL-1 from activated T-cell lysates were captured on a BIAcore CM5 chip by using mAbs Hermes-3 and KPL-1, respectively. To study the ability of immobilized CD44/Hermes-3–mAb and PSGL-1/KPL-1–mAb complexes to interact with E-Ig, we injected 100-nM E-Ig in the presence of 5-mM EDTA or 1-mM Ca^2+^ at 50-mM NaCl. The accumulated number of E-Ig response units on the complexes during both E-Ig injection and buffer washing was measured and subtracted from the non-specific interaction with the isotype controls. Our data show that both proteins are able to bind E-Ig. Again, the specificity of the interaction was confirmed by the loss of binding when EDTA was added (Figure 3B, *upper panels*).

We next aimed to measure and compare the affinity and kinetics of CD44/HCELL and PSGL-1 to bind E-Ig. To achieve this, we used the steady-state model to determine the *K*_D_, which required injection of serial dilutions of E-Ig over the stably immobilized CD44/HCELL and PSGL-1 ligands. These studies were performed in buffers containing 150-mM NaCl to resemble the salt concentration in the circulation. As illustrated in Figure 3B (*lower panels*), E-Ig interacted with its ligands in a dose-dependent manner and we determined the *K*_D_ for E-Ig binding to CD44/HCELL (777 ± 40 nM) and PSGL-1 (467 ± 95 nM) to be comparable. Following the final E-Ig injection and during the buffer washing step, we determined the apparent dissociation rate constant (*k*_off-apparent_) to be 4.9 × 10^−5^ ± 2.1 × 10^−5^ s^−1^ for CD44/HCELL and 25 × 10^−6^ ± 4.7 × 10^−6^ s^−1^ for PSGL-1 (Figure 3B, *lower panels*). Thus, using Eq. 1 in Supplementary Material, the estimated apparent association rate constant (*k*_on-apparent_) was 61 ± 24 M^−1^ s^−1^ for CD44/HCELL and 52 ± 5.5 M^−1 ^s^−1^ for PSGL-1 suggesting no statistically significant difference between the dissociation rate or the association rate of CD44/E-Ig and PSGL-1/E-Ig (*P* = 0.3 and 0.8, respectively). Overall, these results demonstrated that both PSGL-1 and CD44/HCELL isolated from activated human T-cells bound E-Ig with similar kinetics which is consistent with the affinity and kinetics evaluated for CD44/HCELL and PSGL-1 from the human leukemic progenitor cell line KG1a with E-Ig (40). These results are further supported by the data we obtained from the blot rolling assay, in which both ligands were able to support the rolling of CHO-E cells with similar binding velocity.

To expand upon the limited information available on the stoichiometry of E-selectin binding to its ligands, we proceeded to quantify the stoichiometry of recombinant E-selectin binding to CD44/HCELL and PSGL-1 in 150-mM NaCl buffer. To achieve this, we calculated the observed maximum number of response units using Eqs 2 and 3 in Supplementary Material. While only 60 ± 23% (mean ± SEM; *n* = 4) of captured CD44/HCELL was appropriately oriented and/or glycosylated to bind E-Ig, around 221 ± 72% (mean ± SEM; *n* = 4) of PSGL-1 could bind indicating that a minimum of two E-selectin dimers interacts with a dimer of PSGL-1. Collectively, these results indicate that although the intrinsic binding kinetics of both ligands are similar, variation could occur due to differences in the avidity of their individual binding sites, the degree of posttranslational modifications on the ligands, and/or the expression level of each ligand. Indeed, the CD44 antibodies used for these experiments are not specific to any specific glycoform of CD44 so they are likely capturing a mixture of glycoforms of CD44 and the HCELL glycoform is able to bind E-Ig, while the others do not as previously discussed (40).

### Both CD44/HCELL and PSGL-1 Are Essential for E-Selectin Binding of Human Activated T-Cells

**Figure 4:**
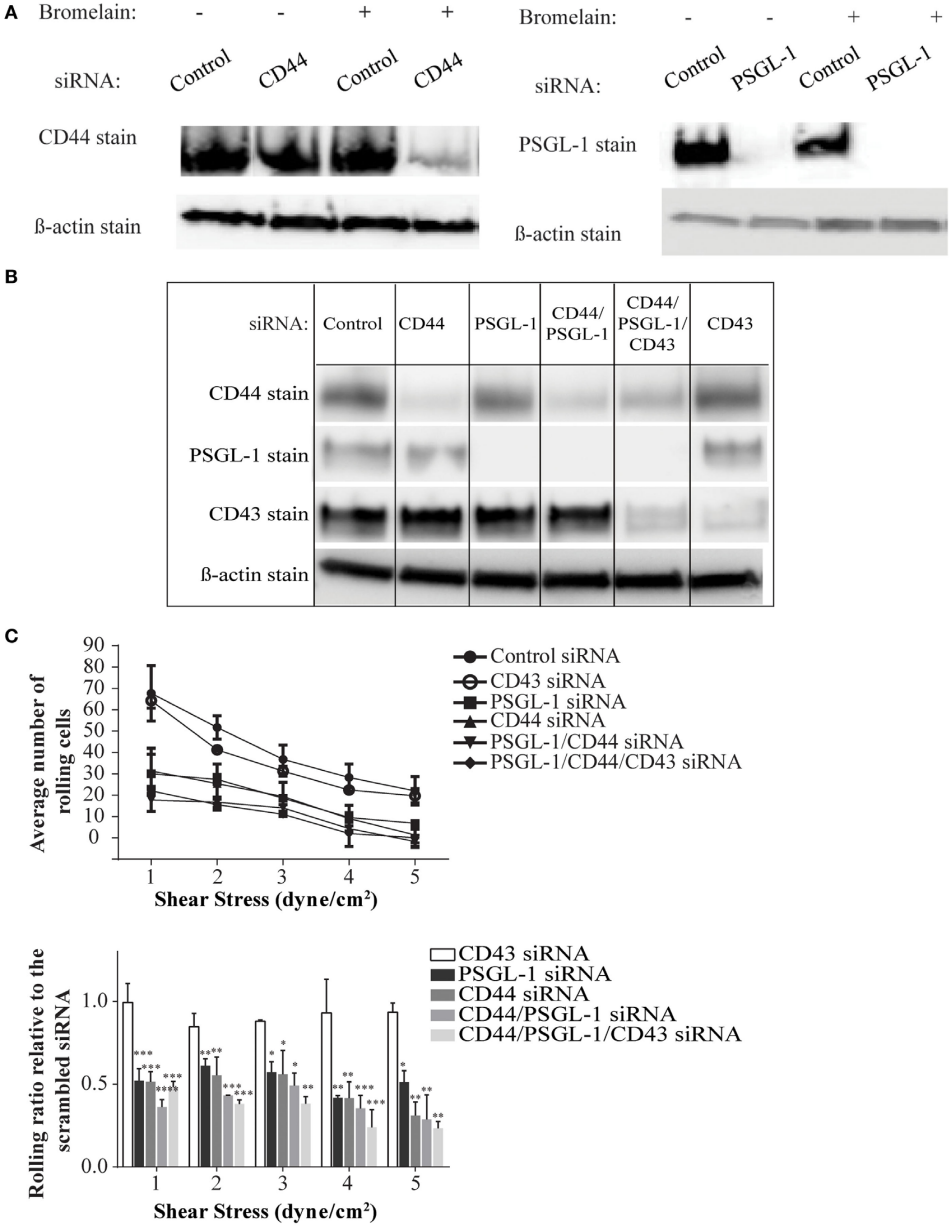
**CD44/HCELL collaborates with P-selectin glycoprotein ligand-1 (PSGL-1) to confer the optimal rolling of activated human T-cells over E-selectin expressing monolayers**. Small interfering RNA (siRNA) knockdown technologies were used to effectively decrease the expression of various E-selectin ligands on activated human T-cells. To evaluate the effect of bromelain treatment on the transfection efficiency, the cells were treated with bromelain and then transfected with scrambled control siRNA, CD44 siRNA, or PSGL-1 siRNA. After 24 h, live cells were collected to evaluate transfection using Western blot analysis. **(A)** The knockdown of CD44 (*left panel*), but not PSGL-1 (*right panel*), was enhanced by bromelain treatment prior to the siRNA transfection. **(B)** The specificity of gene silencing was demonstrated by Western blot analysis. The removal of one ligand did not affect the expression of other ligands. **(C)** Transfected cells were perfused over CHO-E cell monolayers for 30 s at 1 dyn cm^−2^ wall shear stress, and then detachment assays were performed by a stepwise increment of the shear stress every 30 s. The average number of rolling cells at the end each shear stress is depicted. A significant difference was observed between scrambled control cells and all other knockdown transfections except CD43 knockdown. No major differences were observed between cells treated with CD44, PSGL-1, double (CD44 and PSGL-1), or triple (CD44, PSGL-1, and CD43) siRNAs. Data are mean ± SEM; *n* = 3. *p*-Values comparing the different groups versus scrambled control siRNA are indicated: *p < 0.05, **p < 0.01, and ***p < 0.001.

To date, antibodies that block specific ligand binding to E-selectin have not been described, making it difficult to compare the contribution of individual ligands to the overall E-selectin binding of a cell. Furthermore, differences in the expression levels of different ligands and their immuno-purification efficiency pose challenges to comparative analysis. Moreover, no gene-targeted silencing studies of E-selectin ligands have been conducted on primary activated human T-cells or on any other primary human cells to our knowledge. To this end, we used a siRNA-mediated knockdown approach to dampen the expression of CD44 (i.e., HCELL) and PSGL-1 either individually or in tandem and assessed effects on E-selectin-mediated cell rolling. Individual cell membrane proteins exhibit different turnover rates; therefore, we predicted that these proteins persist even after successful knockdown of their mRNAs and elicit a false impression of incomplete silencing. To circumvent this issue, we used bromelain to remove cell surface proteins prior to transfection. Western blot analysis verified that this procedure resulted in a more thorough knockdown of the cell surface proteins, which was more pronounced on the knockdown of CD44 than on the PSGL-1 knockdown (Figure 4A), likely due to a variation in the turnover rates between the two ligands. As shown in a representative Western blot in Figure 4B, effective knockdown was observed in the single knockdowns (CD44, PSGL-1, and CD43), the double knockdown (CD44 and PSGL-1), and the triple knockdown (CD44, PSGL-1, and CD43). Furthermore, the expression level of non-targeted ligands remained consistent among all treatments confirming the specificity of each siRNA.

To investigate the impact of ligand silencing on E-selectin-mediated rolling, activated human T-cells transfected with CD44, PSGL-1, double, CD43, or triple siRNAs were perfused over a monolayer of CHO-E cells under several shear stress conditions (1–5 dyn cm^−2^). Cells transfected with a scrambled control siRNA maintained high numbers of rolling interactions on CHO-E cells at all shear stress rates; however, in the absence of CD44/HCELL and/or PSGL-1, a marked reduction in the number of rolling cells at shear stress ≥1 dyn cm^−2^ was observed (Figure 4C; Videos S3–S8 in Supplementary Material). Knockdown of both ligands did not result in a synergistic decrease in rolling behavior, beyond that observed in the individual knockdowns, strongly suggesting that the presence of both CD44/HCELL and PSGL-1 was essential to confer rolling over CHO-E cells (Figure 4C; Videos S3–S8 in Supplementary Material). Unlike CD44/HCELL and PSGL-1, the knockdown of CD43 did not affect the number of rolling cells. Moreover, loss of CD43 from the triple knockdown did not result in further decreases in the number of rolling cells compared with the number with double knockdown, indicating that CD43 minimally contributed to E-selectin-mediated rolling. The function of CD43 in T-cell migration is conflicting: some data implicate it as an anti-adhesive (42–44) molecule while others as a pro-adhesive one (19, 20, 45, 46); however, in this study, we could not confirm the pro-adhesive role of CD43 on activated human T-cells mainly comprised of a Th2 phenotype (Table S2 in Supplementary Material). Furthermore, in the absence of both of these ligands, some binding to E-selectin still persists, likely due to glycolipids (47), existence of low levels of CD44 (Figure 4B), and/or other minor glycoprotein ligands as outlined in our mass spectrometry study (Table 1).

### CD44/HCELL on T-Cells Isolated from Psoriatic Patients Binds E-selectin

**Figure 5:**
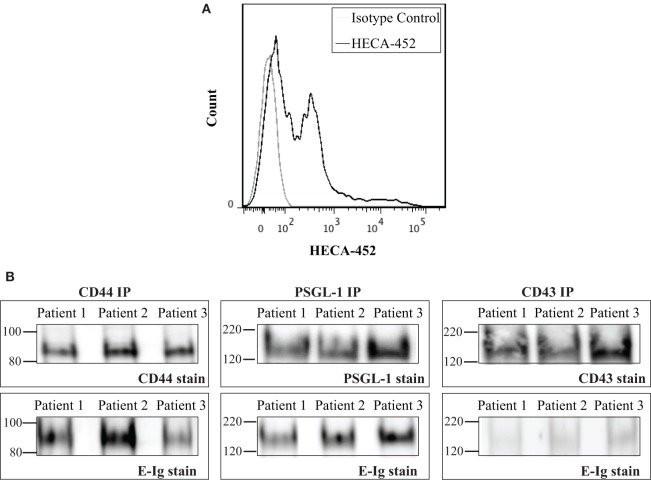
**CD44/HCELL on T-cells from psoriatic patients binds E-selectin**. **(A)** Expression of sialofycosylated glycans on T-cells isolated from psoriatic patients. After isolation of CD3^+^ cells from psoriatic patients (representing mixed subsets as illustrated in Table S2 in Supplementary Material), we performed flow cytometry analysis to confirm the expression of sialofycosylated glycans using HECA-452 antibody. **(B)** CD44, P-selectin glycoprotein ligand-1 (PSGL-1), and CD43 were immuno-purified from T-cells obtained from three psoriatic patients and then samples were prepared for Western blot analysis and blotted for each ligand (*upper panels*) and for E-Ig (*lower panels*). Note that CD44/HCELL and PSGL-1 exhibit strong binding to E-Ig, while CD43 binding was very weak.

The aforementioned results showed that CD44/HCELL from *in vitro*-activated T-cells possess E-selectin-binding activity. Next, we examined whether the ability of CD44/HCELL to bind E-selectin would be preserved under *in vivo* stimulation. To this end, we isolated circulating T-cells from patients suffering from the chronic skin inflammatory disease, psoriasis. Many studies have implicated that E-selectin plays a key role in the excessive infiltration of memory T-cells to the skin that manifests as psoriasis (6, 48–50). Moreover, several studies have confirmed the importance of circulating T-cells bearing the HECA-452 antigenic determinant in the clinical manifestation of psoriasis (51, 52). We confirmed the expression of HECA on circulating T-cells isolated from psoriatic patients using flow cytometric analysis (Figure 5A). The percentage of T-cells expressing HECA was significantly higher in psoriatic patients than in healthy donors (*P*-value = 0.007), 36 ± 4.3% (mean ± SEM; *n* = 4) versus 10.6 ± 3% (mean ± SEM; *n* = 3), respectively. We next immuno-purified E-selectin ligands from T-cells isolated from psoriatic patients and tested their interaction with E-selectin. As illustrated in Figure 5B, CD44/HCELL and PSGL-1 bound E-selectin. Similar to our *in vitro*-activated T-cell results, a very weak interaction was observed between CD43 and E-selectin. Overall, this study identifies CD44/HCELL as an E-selectin ligand on activated human T-cells and suggests that it may play an important role in the regulation of T-cell migration and/or trafficking in skin diseases.

## Discussion

CD44 has a well-established role in T-cell migration *via* its interactions with HA (30) and the integrin VLA-4 (53). Here, we provide compelling evidence that CD44/HCELL expressed by *in vitro*- and *in vivo*-activated human T-cells can mediate T-cell interactions with E-selectin. We showed that CD44/HCELL bound E-selectin in a transient and specific manner and that this interaction supported cell rolling under physiological shear stress conditions similar to the well-described E-selectin ligand PSGL-1. In fact, our data demonstrate that CD44/HCELL mediates this action through cooperation with PSGL-1.

Several E-selectin ligands have been identified on mature myeloid cells, hematopoietic progenitor cells, cancerous cells, and lymphocytes (18, 27, 42–44, 54). To date, only two of these ligands, PSGL-1 and CD43, are reported to be E-selectin ligands on activated human T-cells (19, 20). Studies in mice showed that a concomitant deficiency of these ligands did not completely abolish E-selectin-mediated T-cell rolling (20), suggesting the requirement of another E-selectin ligand(s). Moreover, reports on the role of CD43 in T-cell migration show conflicting results: some works propose its anti-adhesive role (42–44, 54) and others propose its pro-adhesive role (19, 20, 45, 46, 55). Therefore, we performed a mass spectrometry-based analysis that showed additional E-selectin ligands are present on activated human T-cells, such as CD44 as well as some other ligands that require confirmation for their ability to bind E-selectin. Herein, we characterize the role of CD44 in E-selectin-mediated T-cell rolling, and in future work, we plan to determine the relevance of the remaining candidate ligands.

Previous works identified that CD44 is an E-selectin ligand on several different types of cells, such as HSPCs, neutrophils, mouse Th1 cells, breast cancer cells, colon carcinoma cells, and melanoma (24, 26, 28, 29, 41, 56). Actually, a specialized glycoform of CD44 is able to bind E-selectin and based on the extensive biochemical characterization of this binding, the E-selectin binding form of CD44 became known as HCELL. In a number of recent reviews, HCELL is described as a sialofucosylated glycoform of CD44, originally found on HSPCs, that acts as an E-selectin and/or L-selectin ligand (57, 58). CD44 isolated from activated human T-cells stains for antibodies that recognize sialofucosylated glycans (mainly HECA-452, CSLEX-1, and KM93, Figure S3 in Supplementary Material). However, the signal we detect with CD44 is weaker than the PSGL-1 signal. This weaker signal with HECA-452 mAb might explain why CD44 was missed in previous studies on activated human T-cells in which researchers were focusing on identifying ligands that displayed strong HECA-452-staining signal (19). Similar to earlier reports, the importance of *N*-glycans in mediating the interaction of E-selectin binding to CD44/HCELL (26, 27, 40) on primary HSPCs was also demonstrated here on activated human T-cells. Interestingly, previous work has shown that *N*-glycosylation is predominant on CD44s, whereas *O*-glycosylation is more predominant on CD44v (24). This is consistent with our finding that E-selectin binding form of CD44/HCELL on activated human T-cells is indeed CD44s.

In an effort to characterize the binding affinity and kinetics between the dimer form of E-selectin (E-Ig) and CD44/HCELL or PSGL-1 from activated human T-cells, we used the real-time BIAcore technique. Our results were consistent with a previous study that evaluated the affinity and kinetics between E-Ig and CD44/HCELL or PSGL-1 from the human leukemic progenitor cell line KG1a (40). Although both ligands bound to E-Ig with a similar affinity at a physiological salt concentration of 150-mM NaCl, the ligands bind to E-Ig with different valences: one dimer of PSGL-1 interacted with a minimum of two dimers of E-selectin and almost half of the CD44 bound E-selectin. The remaining CD44 may be inadequately stained with HECA or inappropriately oriented on the surface of the CM5-Chip to interact with E-selectin. Since the CD44 antibodies are not specific for the HCELL glycoform, at least partially inadequate glycosylation of the remaining CD44 is likely a reason for the observed stoichiometry as previously noted (40). The finding that both CD44/HCELL and PSGL-1 bound to E-selectin with similar kinetics was further supported by the data we obtained from the blot rolling assay, in which both ligands were able to support the rolling of CHO-E cells with similar binding velocity.

Using a knockdown approach on primary human T-cells, we dampened the expression of CD44/HCELL, PSGL-1, and CD43 either individually or together to determine the importance of each ligand on E-selectin binding. We found that the success of CD44 (and HCELL) transfection was dependent on treating activated cells with bromelain prior to the transfection to remove any previously expressed proteins. While bromelain treatment was not required for effective knockdown of PSGL-1, we attributed the more thorough knockdown with the treatment to a variation in the turnover rates between the two ligands. The parallel plate flow assay on transfected cells showed that the loss of either CD44/HCELL or PSGL-1 had an adverse impact on the rolling capacity of the activated T-cells over CHO-E cells. The simultaneous knockdown of both ligands did not result in a synergistic decrease in rolling behavior, which strongly suggests that both CD44/HCELL and PSGL-1 collaborate to confer efficient rolling of activated human T-cells over CHO-E cells. Furthermore, in the absence of both CD44/HCELL and PSGL-1, we continued to observe residual binding to E-selectin, which we attributed to the availability of glycolipids (47, 59) as well as other minor glycoprotein ligands. Potentially, some of these minor ligands might have been detected by the mass spectroscopy studies we conducted (Table 1; Table S1 in Supplementary Material). Further studies are required to assess the interactions of E-selectin with these candidate ligands under physiological shear stress conditions.

We could not confirm the pro-adhesive role of CD43 on activated human T-cells. The removal of CD43 in CD43 siRNA transfected cells did not affect the rolling capacity when compared with scrambled siRNA transfected cells. In addition, the removal of CD43 in the triple knockdown did not significantly decrease E-selectin-mediated rolling beyond the decrease we noticed in the CD44/HCELL and PSGL-1 double knockdown. Furthermore, the minimal contribution of CD43 to E-selectin binding in chronic skin disease was evident when immuno-purified CD43 from psoriatic patients retained an extremely weak interaction with HECA-452 and E-selectin. Detection of binding between E-selectin and CD43 in both the mass spectrometric and Western blot analyses could be explained by the nature of these assays: both are static, unlike parallel plate flow chamber assays, blot rolling assay, and BIAcore (data not shown), which rely on flow. Thus, it is possible that the application of mechanical load on CD43 in the later assays caused shear-induced conformational changes (60) that buried E-selectin-binding sites on CD43 and disrupted its binding activity. In addition, it is unclear whether the expression of HECA-452-reactive epitope on any protein by itself is sufficient to qualify it as a functional E-selectin ligand. Moreover, although CD43 may not specifically contribute to E-selectin-binding interactions, its importance in binding to other potential ligands such as ICAM-1 and galectin-1 (61, 62) should not be overlooked given that these ligands are expressed by inflamed endothelium and that the administration of human recombinant galectin-1 can inhibit leukocyte rolling and extravasation (63).

E-selectin-specific inhibitors are currently being investigated for their positive effects on acute myeloid leukemia and sickle cell disease (64–66). By inhibiting selectin interactions, GMI-1070 can decrease leukocyte adhesion and recruitment to inflamed tissues in various disorders, such as the inflamed venules in sickle cell disease, an essential event initiating vaso-occlusion in this disease. In animal models, GMI-1070 also appears to inhibit the homing of multiple myeloma cells to the bone marrow and improves the efficacy of the proteasome inhibitor, bortezomib, which is currently used to treat the disease (67). This is an attractive therapeutic target in autoimmune inflammatory diseases such as rheumatoid arthritis and psoriasis in which the expression of E-selectin has been shown to be responsible for the pathological accumulation of active immune cells such as lymphocytes (8, 68). For example, in psoriasis patients, the administration of a pan-selectin inhibitor (Bimosiamose) resulted in a reduction of lymphocyte infiltration and an overall clinical improvement (69). However, the long-term use of pan inhibitors might interfere with vital interactions relating to homeostasis such as the migration of HSCs to the bone marrow (27, 70) and in immune cell migration to infected tissues (9, 71). Therefore, targeting specific E-selectin ligand(s) may be a more attractive treatment strategy.

To the best of our knowledge, limited studies have looked into identifying all the E-selectin ligands involved in the recruitment of T-cells in psoriasis and/or any other inflammatory skin disorders. Here, we show evidence that both CD44 (and specifically HCELL) and PSGL-1 immuno-purified from psoriatic patients interacts with E-selectin. Based on these data, we can envision targeting these ligands. Indeed, CD44 blocking antibodies caused a marked reduction in leukocyte infiltration in experimental models of inflammatory diseases (72, 73). In those studies, the apparent infiltration was mainly attributed to the blocking of HA-mediated leukocytes migration. However, neither the inhibition of T-cells nor the contribution of E-selectin-mediated rolling was assessed as the role of CD44/HCELL as an E-selectin ligand on activated human T-cells was not yet discovered. We believe that targeting the E-selectin-binding site on CD44/HCELL could be a viable option to treat skin diseases, such as psoriasis, for a number of reasons relating to homeostasis. Being unable to interact with P-selectin, blocking antibodies against CD44/HCELL can interfere with E-selectin-mediated cell rolling while sustaining the proper immune defense where its interaction with P-selectin is crucial. Moreover, blocking CD44/HCELL interactions with E-selectin may have minimal effects on stem cell homing (74) since many ligands (unpublished data from our lab) could compensate for inhibition of CD44/HCELL binding to E-selectin.

## Experimental Procedures

### Cells

Frozen normal peripheral blood mononuclear cells isolated from subjects with and without psoriasis were ordered from AllCells (CA, USA). Naïve Pan T-cell Isolation Kit and Pan T-cell Isolation Kit (Miltenyi Biotec) were used to isolate T-cells. For activation, 10^6^ cells ml^−1^ of CD3^+^ cells were incubated with 5 μg ml^−1^ of immobilized anti-CD3 (clone: OKT3, eBioscience) in RPMI media, 10% FBS, 1 μg ml^−1^ anti-CD28 (BD Bioscience), and 100 U ml^−1^ of IL-2 for 36 h. Then, cells were collected and cultured for 2 days with 100 U ml^−1^ of IL-2 only.

### Flow Cytometry Analysis

CD25 was used as a T-cell activation marker. The presence of E-selectin ligands on T-cells was determined by assaying the binding of recombinant mouse E-selectin/CD62E Fc chimera (E-Ig) (R&D). First, cells were washed and resuspended in HBSS containing 5% FBS, 25-mM HEPES, and 2-mM CaCl_2_ or 10-mM EDTA (negative control) and then stained for 30 min at 4°C with E-Ig followed by biotin-conjugated mouse anti-human IgG antibody; stained cells were recognized by PE-labeled streptavidin. Binding was detected using the FACSCanto ΙІ and analyzed using FlowJo software.

### Deglycosylation Assay

Immuno-purified proteins were treated with 20 mU ml^−1^ PNGase (NEB), 0.1 U ml^−1^ sialidase (Roche), or 120 μg ml^−1^ of OSGE (Cedarlane); treatments with PNGase or OSGE were performed for 4 h at 37°C, and the sialidase treatment was done in HBSS with 5-mM CaCl_2_ for 3 h at 37°C. As a control, each treatment was performed under the same conditions with no added enzymes.

### Blot Rolling Assay

This assay was carried out as previously reported (75, 76). Briefly, CHO cells expressing E-selectin (CHO-E) were harvested with 10-mM EDTA, washed, and resuspended in HBSS (135-mM NaCl) at 10^7^ cells ml^−1^. Western blot membranes of immuno-purified PSGL-1 or CD44/HCELL were stained with HECA or 2C5 antibody, respectively, and rendered transparent using HBSS, 2-mM Ca^2+^, and 10% glycerol. Next, the blots were placed in the parallel plate flow chamber, 10^6^ cells were perfused into the chamber at a shear stress rate of 0.5 dyn cm^−2^, and rolling cells were captured on videotape. The number of rolling cells and the cell displacement on the *Y*-axis over time (velocity, micrometers per second) for both proteins were determined using Imaris V7 software (Bitplane). Only cells with a minimum diameter of 10 μm and with minimum threshold intensities were included in the analyses. To count the number of rolling cells, those that moved less than four frames or had a total displacement of less than 10 ± 3 μm were considered non-specific and eliminated from the analysis. To measure the velocity, we plotted the position of each rolling cell at any one field (micrometers) over time (seconds) and then calculated the fitted slope as an indicator of the average velocity.

### BIAcore Analysis

The assay was carried out as described previously (40); mAbs against CD44 and PSGL-1 were directly immobilized over CM5 sensor chip by amine coupling to capture the native proteins from the fresh T-cell lysate. Details are supplied in experimental procedures in Supplementary Material.

### Gene-Targeted Knockdown of CD44, PSGL-1, and CD43

T-cells were activated as described above, and on Day 2 of activation, the cells were collected and treated for 7 min at 37°C with 1 mg ml^−1^ of bromelain (Sigma) in RPMI media with 10% FBS to preferentially cleave the carbonyl ends of lysine, alanine, tyrosine, and glycine. The cells were then washed three times in media and prepared for transfection. To knock down CD44, we used 50 pmol of the siRNA sequences s2681 and s2682; to knock down PSGL-1, we used 100 pmol of s12688; and to knock down CD43, we used 140 pmol of s13368 (Silencer Select, Life Technologies). Negative control no. 1 was used as the scrambled sequence for the transfection (Silencer Select, Life Technologies; 4390843). The cells were transfected according to the company protocol (Lonza) using the P3 Primary Cell 4D X Kit (V4XP). After 24 h of transfection, dead cells were removed by Ficoll separation and live cells were collected for parallel plate flow chamber assay and Western blot analysis.

### Parallel Plate Flow Chamber Assay

Transfected cells were resuspended in HBSS/2-mM CaCl_2_ (10^6^ cells ml^−1^) and perfused over a confluent monolayer of CHO-E cells at shear stresses starting from 0.3 dyn cm^−2^ for 2 min followed by gradual increases every 30 s from 1 to 5 dyn cm^−2^. Experiments were observed in real time and videotaped for analysis. Statistical analysis was performed using one-way ANOVA followed by multiple comparisons and a *Tukey’s* test for correction (GraphPad Prism).

### Online Supplementary Material

Detailed methods and representative videos of the cell rolling experiments shown in Figure 1 and the blot rolling assays in Figure 3 are available in experimental procedures in Supplementary Material.

## Author Contributions

AJA designed, performed, and analyzed experiments and wrote the manuscript. AFA helped in designing and conducting the cell-rolling experiments, maintaining cancer cell lines, and discussing the results. JM designed and analyzed experiments and wrote the manuscript.

## Conflict of Interest Statement

The authors declare that the research was conducted in the absence of any commercial or financial relationships that could be construed as a potential conflict of interest.

## Supplementary Material

Videos S1 and S2 **The videos show the data from blot rolling assays that were performed on immuno-purified CD44 (Video 1) and PSGL-1 (Video 2) from activated human T-cells**. Refer to Figure 3A. Videos were recorded and analyzed at 1 frame s^−1^ but for presentation purposes are displayed at 5 frames s^−1^.

Videos S3–S8 **Examples of rolling activated human T-cells transfected with various knockdown siRNAs to assess the effect of ligands on migration over E-selectin expressing CHO cell monolayers**. Video 3, scrambled vector control; Video 4, CD44 siRNA; Video 5, CD162 (PSGL-1) siRNA; Video 6, double siRNA; Video 7, triple siRNA; and Video 8, CD43 siRNA. Refer to Figure 4C. Videos were recorded and analyzed at 1 frame s^−1^ but for presentation purposes are displayed at 5 frames s^−1^.

## Abbreviations

E-Ig, recombinant E-selectin–Ig chimera; HA, hyaluronic acid; HSPCs, hematopoietic stem/progenitor cells; HCELL, hematopoietic cell E- and/or L-selectin ligand; mAbs, monoclonal antibodies; PSGL-1, P-selectin glycoprotein ligand-1; siRNA, small interfering RNA; sLex/a, sialyl Lewis x/a.
