# Supplementary material for: An Analysis of Trafficking Receptors Shows that CD44 and P-Selectin Glycoprotein Ligand-1 Collectively Control the Migration of Activated Human T-Cells
Source: Front Immunol. 2017 May 3;8:492. doi: 10.3389/fimmu.2017.00492 (PMC5413510; doi:10.3389/fimmu.2017.00492)
Supplement: Supplementary file 9 [file Data_Sheet_1.PDF]

Supplementary Material

An analysis of trafficking receptors shows that CD44 and PSGL-1 collectively control the migration of activated human T-cells

Amal J. Ali[1], Ayman F. Abuelela[1] and Jasmeen S. Merzaban[1†]

[1]King Abdullah University of Science and Technology (KAUST), Division of Biological and Environmental Sciences and Engineering (BESE), Thuwal 23955, Saudi Arabia

1 Supplementary Methods

Antibodies and recombinant proteins

The following primary antibodies were purchased from BD Bioscience: anti-human CD25 (BD, $MsIgG_1$), anti-human CD44 (R&D, clone: 2C5, $MsIgG_{2a}$), anti-human CD43 (BD, clone: L60 and 1G10, $MsIgG_1$), anti-human PSGL-1 (clone: KPL-1, mIgG1), anti-CLA (BD, Rat-IgM), anti-CD15s (BD, MsIgM) and PE-labeled Streptavidin. We also used anti-human PSGL-1 (clone: C-1, mIgG2a) from Santa Cruz. Secondary antibodies were purchased from Thermo Fisher Scientific: HRP anti-human IgG, biotin-conjugated mouse anti-human IgG, and HRP-goat anti-mouse IgG. In addition, we used HRP goat anti-human IgG from southernbiotech. To separate $CD4^+$ and $CD8^+$ cells, cells were labeled with either FITC-conjugated anti-CD4 mAb (Miltenyi Biotec) or APC-conjugated anti-CD8 mAb (BD) and then separated by anti-FITC or anti-APC microbeads (Miltenyi Biotec), respectively using autoMACS cell-sorting machine. Recombinant human P-selectin/ IgG chimera (P-Ig) or recombinant human L-selectin/ IgG chimera (L-Ig) were bought from R&D. PE anti-human IL-4 (Clone: 8D4-8), Pacific Blue anti-human IL-17A (Clone: BL168) and APC anti-Human IFN-Gamma (Clone B27) were bought from Biolegend. Fixation/Permeabilization Solution Kit was bought from BD. FITC anti-Human CD44v7 and FITC anti-Human CD44v7/8 were bought from

ebioscience. Mouse anti-human CD44v10 (clone: VFF-14), APC mouse anti-human CD44v3 and PE mouse anti-human CD44v4/5 were bought from Fisher Scientific. FITC mouse anti-human CD44v6 (clone: VFF-7) were bought from Invitrogen. Purified mouse anti-human CD44v6 (clone: 2F10) were bought from R&D. Rat anti-human CD44v9 were purchased from Abnova (clone: RV3).

Immunoprecipitation and Western blot

Cells were lysed using CHAPS lysis buffer (FIVEphoton biochemicals) or 1% Triton X-100 (Fisher scientific) in 150-mM NaCl, 50-mM Tris-Base, at pH 7.4 with 1-mM phenylmethanesulfonyl fluoride and a protease inhibitor cocktail (Roche) at 4^0C. Next, the lysate was pre-cleared and incubated overnight with recombinant E-Ig (5μg) in the presence of 5-mM $CaCl_2$ or with CD44 mAbs (clone: IM7 from Biolegend and 515 from BD Bioscience 2μg/each) or with PSGL-1 mAb (clone: KPL-1 from Biolegend and clone 1.BB.238 from Santa cruz) and each with 50μl of Dynabeads® Protein G. Next, the supernatant was collected to verify the efficiency of the IP while the remaining bead-antibody-antigen complex was washed and then resuspended in an adequate amount of lysis buffer, NuPAGE LDS sample buffer (Invitrogen) and 10% β-mercaptoethanol, followed by protein elution by heating for 10min at 70°C. The immuno-purified proteins were subjected to SDS-PAGE and transferred to a PVDF membrane. The resulting membrane was blocked using Tris-buffered Saline with Tween-20 (Cell Signaling Technology) containing 5% non-fat milk and then incubated with the recombinant E-Ig, anti-human CD44 (clone: 2C5 (R&D) or Hermes-3 (Abgent)), or anti-human PSGL-1 (KPL-1, BD). Next, the membranes were immunoblotted with HRP-conjugated antibodies (Thermo scientific).

Mass spectrometry sample preparation

Proteins were analyzed by mass spectrometry (MS) as previously described (Acestor et al., 2009). Briefly, the immuno-purified E-Ig ligands were separated using SDS-PAGE and the protein bands

were visualized by SYPRO Ruby protein gel stain (Life Technologies). The bands were cut and treated with a trypsin profile IGD kit (Sigma Aldrich). The resulting peptides were extracted using extraction buffer containing 5% acetonitrile, 95% water, and 0.1% formic acid, dried using a speed vacuum, fractionated by Nanoflow LC system, and then analyzed using LTQ Orbitrap. The raw data was converted to the Mascot generic format and searched using the online Mascot database.

BIAcore analysis

Using the BIAcore T-100 system (GE healthcare) as described previously,(AbuSamra et al., 2015) a CM5 sensor chip was activated with a 7-min injection of NHS (*N*-hydroxysuccinimide) and EDC (1-ethyl-3-(3-dimethylamino propyl)carbodiimide hydrochloride) at a 1:1 ratio. We added mAbs against CD44 (Hermes-3 mAb, Abgent) and PSGL-1 (KPL-1 mAb, Biolegend) was directly immobilized by amine coupling to capture the native proteins from the fresh T-cell lysate. To correct against the buffer's bulk refractive index and the non-specific interactions of the lysate, the isotype controls for CD44 and PSGL-1 mAbs were immobilized (mIgG2a and $mIgG_1$, respectively) on the control flow cells. To determine the number of response units (RU) for each immobilized mAb, $20x10^6$ activated human T-cells were lysed in 100µl of 250-mM NaCl, 50-mM Tris-HCL, and 1% triton for 4h; then 200µl of this solution was run over the immobilized mAbs for 700s at 20µl/min. To study E-Ig binding, 30µg/ml of recombinant E-Ig in 50-mM NaCl, 50-mM Tris-HCL, 1% Triton X-100, and 1-mM $CaCl_2$ was injected over the captured proteins for 300s in the presence of 5-mM EDTA or 1-mM Ca^{2+} at 20ul/min; data analyses were performed using BIAcore evaluation software. The actual response unit of E-Ig (RU) with CD44 or PSGL-1 was determined by correcting for the bulk refractive index and subtracting the non-specific interaction of E-Ig with the isotype control. We conducted the kinetic analysis in 150-mM NaCl running buffer. Ligands were immobilized over the chip as described above, and then a series of E-Ig concentrations (0.78-800 nM) were injected at 30µl/min for 300s; a 300s delay between injections was applied and there were no regeneration steps

between injections. To define the equilibrium dissociation constant (K_D), we determined the maximum response unit (RU_{max}) of bound E-Ig after each E-Ig injection, prior to the washing step. We then blotted RU_{max} over the E-Ig serial dilutions and applied a hyperbola fitting (origin software V8). The BIA evaluation software was used to calculate the apparent dissociation rate constant ($K_{off\text{-}apparent}$) of the interaction between mAb/captured-protein complex and E-Ig by fitting the stable phase in the buffer wash. The apparent association constant ($K_{on\text{-}apparent}$) was calculated using equation (1):

$$K_{on-apparent} = \frac{K_{off-apparent}}{K_D}$$

Statistical analyses of the binding kinetics from four independent experiments (n=4) at 150-mM NaCl were performed using parametric-paired *t*-tests (GraphPad Prism V6.05).

The theoretical RU_{max} of E-Ig bound to immobilized ligands (mAb/captured-CD44 or captured PSGL-1 complexes) was calculated based on equation (2):

$$\mathrm{RU}_{max} = \mathrm{RU}_{Ligand} \times \left(\frac{\text{Molecular weight of analyte}}{\text{molecular weight of ligand}}\right) \times \mathrm{Valency}_{Ligand}$$

and the percent activity of the immobilized ligand was calculated from equation (3):

$$\%\ \text{Ligand activity} = \frac{\text{observed RU}_{max}}{\text{theoritical RU}_{max}} * 100\%$$

For example, in one of the four experiments conducted to calculate the ligand activity of CD44 binding to E-Ig at 150-mM NaCl, assuming 1:1 valency between the Hermes-3 mAb and CD44, we found that ~13% of the RU of the mAb (~150 kDa) captured 263.2 RU of the CD44 (~85 kDa). In total, 960.5 ligand response units (RU_{ligand}) were captured; 613.1 RU are attributed to the 13% surface immobilized mAb and 347.3 RU are associated with captured CD44. Thus, the theoretical RU_{max} from the binding of E-Ig (300 kDa, dimer form of E-selectin) to the 960.5 RU_{ligand} (Hermes-

3/CD44 complex, ~230 kDa), assuming 1:1 valency for CD44/E-Ig interaction, is 1226.1 RU. Our steady state fit data suggest a value of 864.1 RU for the RU_{max}. When we divide the observed RU_{max} value by the theoretical value, it appears likely that only 70.5 % of the captured CD44 are properly oriented to interact with E-Ig or that a maximum of 70.5% captured CD44 are decorated with the appropriate glycosylation required for E-selectin binding. These calculations were also performed for the interaction between PSGL-1-KPL-1 (390 kDa, 149.7 RU_{ligand}) and E-Ig (572.2 fitted RU_{max}), and assuming 2:1 valency of the interaction, we observed around 248% activity of dimer (dimer interaction), indicating that a minimum of two E-selectins interact with a monomer of PSGL-1 at 150-mM NaCl.

Parallel Plate Flow Chamber Assay

Transfected cells were resuspended in HBSS/2-mM $CaCl_2$ (10^6cell/ml) and perfused over a confluent monolayer of CHO-E cells at shear stresses starting from 0.3 dyne/cm^2 for 2min followed by gradual increases every 30s from 1 to 5 dyne/cm^2. The number of rolling cells at the end of each interval was counted and calculated as the ratio relative to the number of cells rolling in the scrambled control siRNA at the same shear stress. For example, at 1 dyne/cm^2, the number of rolling cells in the CD44 siRNA was divided by the number of the rolling cells in the scrambled control siRNA at the same shear stress. Statistical analysis was performed using one-way ANOVA followed by multiple comparison and a *Tukey's* test for correction.

2 Supplementary Figures and Tables

Figure S1: Purity of CD4$^+$ and CD8$^+$ activated T-cells following AutoMACS separation. CD4$^+$ and CD8$^+$ activated T-cells were separated from each other using the Auto-MACS® and the purity of separated cells were assayed by flow cytometry staining of **(A)** the CD4$^+$ population with anti-CD8$^+$ mAb and **(B)** the CD8$^+$ population with anti-CD4$^+$ mAb. More than 90% purity was achieved in both separations.

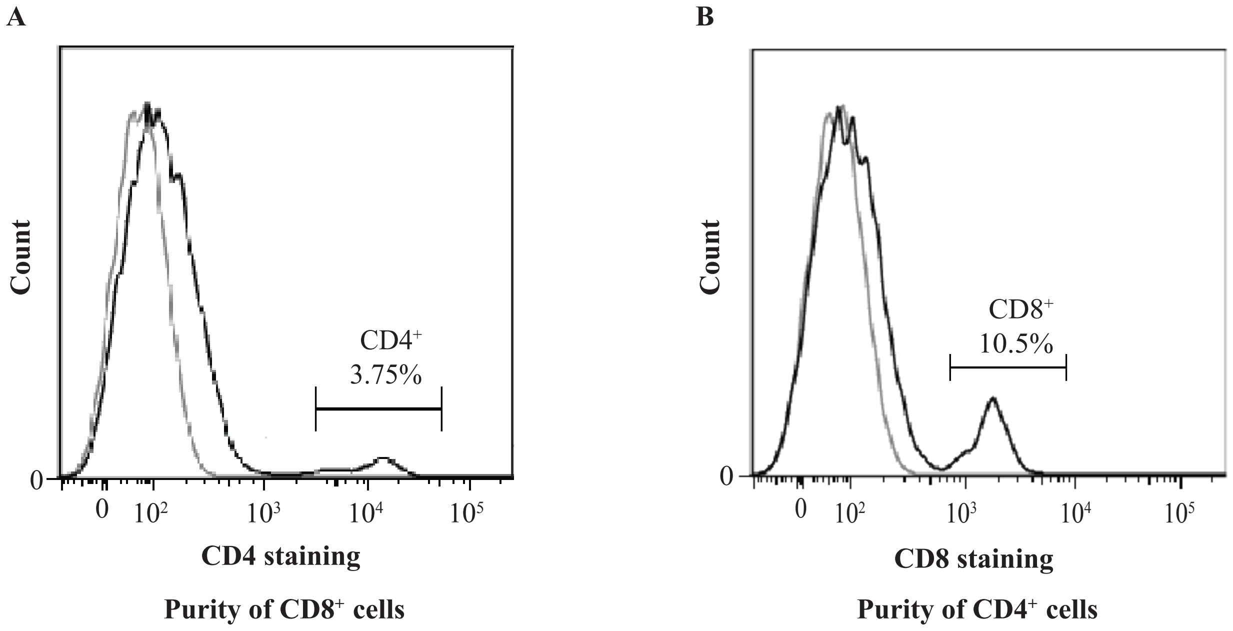

Figure S2: CD44 does not bind P- and L-selectin. CD44 was immuno-purified from activated human T-cells and the purified protein was prepared for Western blot analysis. (**A**) The membrane was stained with anti-CD44 to confirm the identity of the protein. To check whether CD44 bound P- and L-selectin, the membranes were stained with recombinant human P-selectin/IgG chimera (P-Ig) or recombinant human L-selectin/IgG chimera (L-Ig) (**B, C**). CD44 from activated human T-cells does not interact with P-Ig or L-Ig. KG-1a lysate was used as a positive control for P-Ig and L-Ig binding. These are representative blots of *n=3* independent experiments.

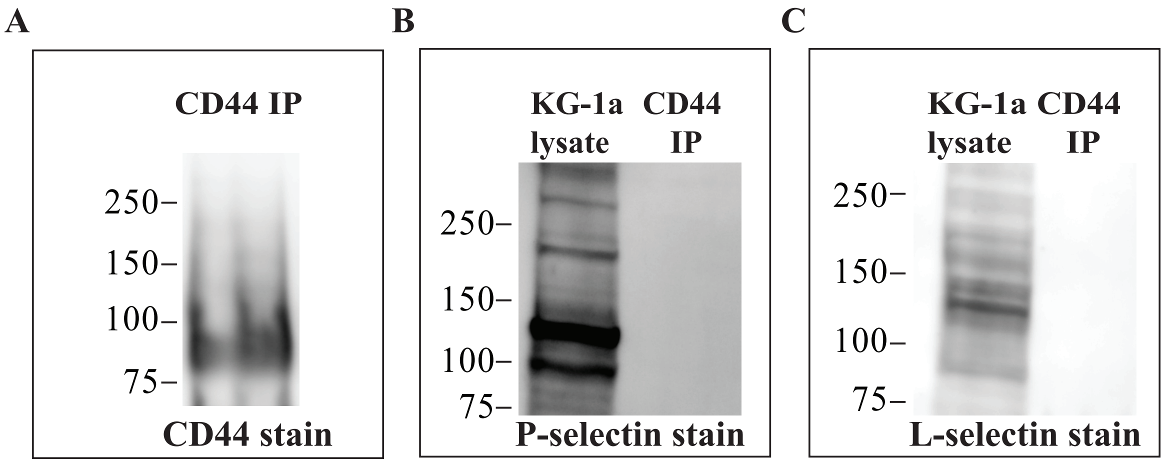

Figure S3: Expression of sialofucosylated glycans on CD44/HCELL. We stained equal amounts of immuno-purified CD44 and PSGL-1 with E-selectin, HECA-452, CSLEX-1 and KM93 antibodies. HECA-452 recognizes sLe^x and sLe^a structures while CSLEX-1 and KM93 recognize sLe^x structures. These are representative blots of *n=3* independent experiments.

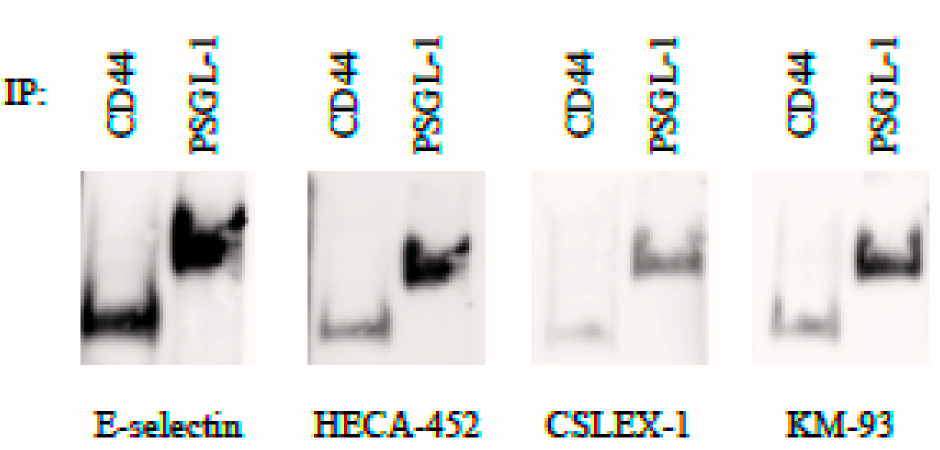

Figure S4: Control immuno-purifications were performed using isotype controls for CD44, PSGL-1 and CD43 mAbs and run alongside immuno-purified CD44, PSGL-1 and CD43. Western blots of these immuno-purified proteins were stained either with secondary antibodies (rabbit anti-mouse IgG) for CD44, PSGL-1 and CD43 antibodies **(A)** or with rabbit anti-human IgG for recombinant human E-selectin/ IgG chimera staining **(B)**. Western blots of immuno-purified samples using isotype controls were stained with recombinant E-selectin and detected using HRP conjugated rabbit anti-human IgG (**C**). We consistently did not detect any non-specific signal from these controls in all blots tested (*n≥10* independent experiments)**.**

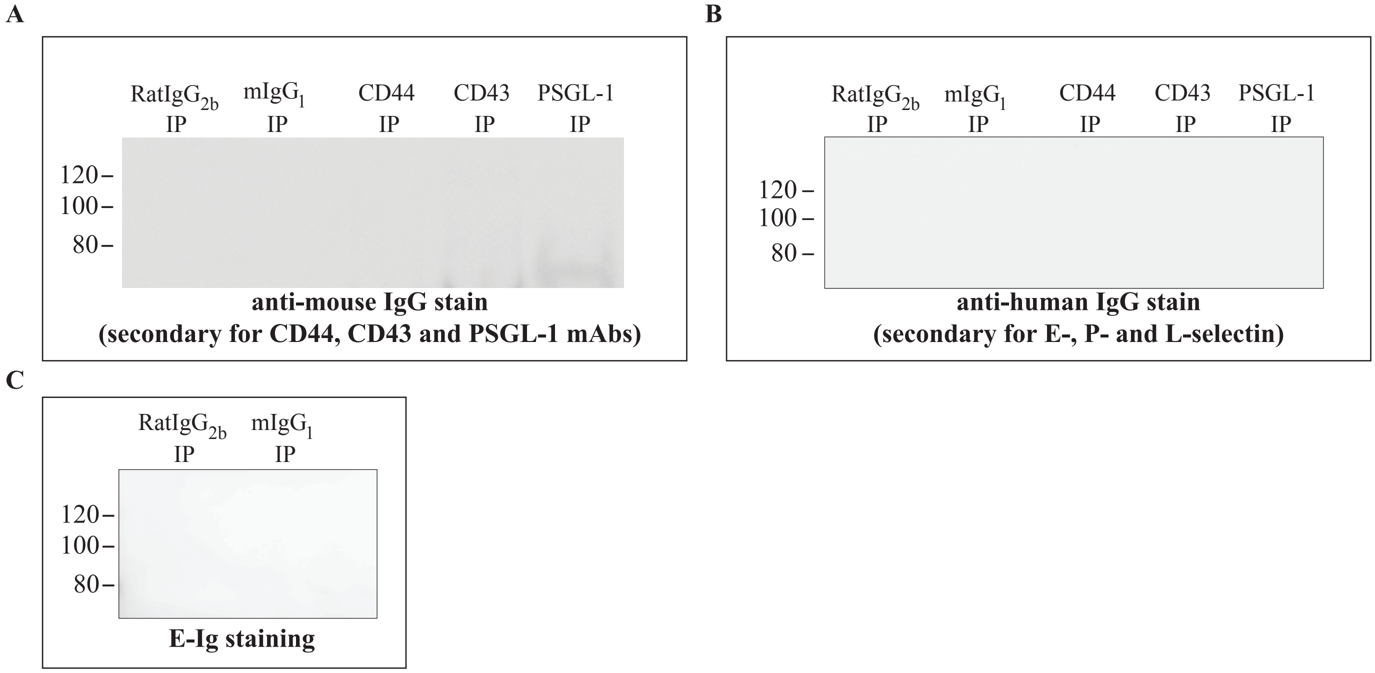

Figure S5: The activity of the CD44v mAbs we used in **Fig. 2** was confirmed by staining MDA-MB-468 cells **(A)** and BT-20 cells **(B)** and analyzing by flow cytometry (Shirure et al., 2015).

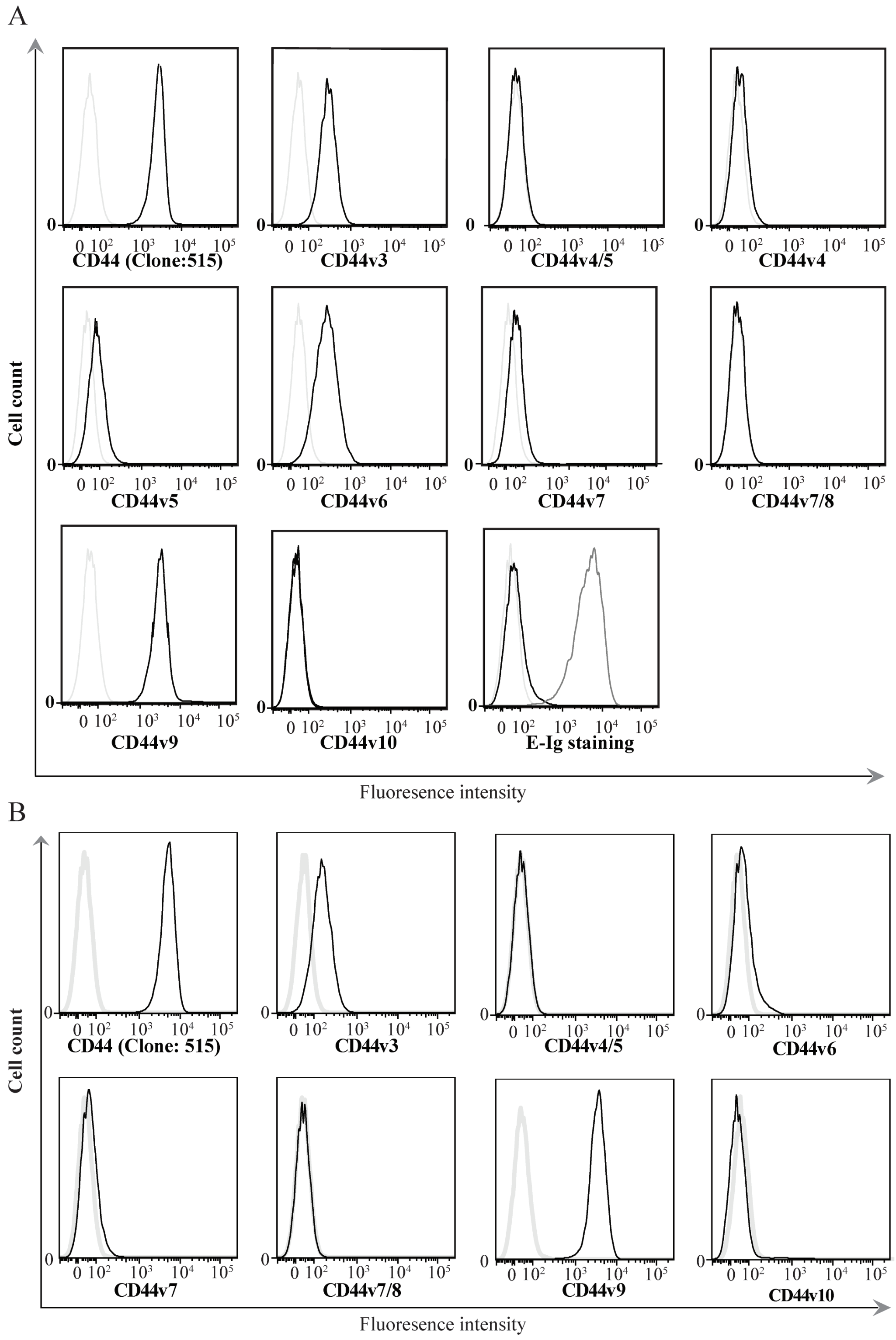

Table S1: E-selectin ligands were immuno-purified from activated human T-cell lysates and the purified proteins were subjected to mass spectrometric analysis for ligand identification. The table lists all the proteins identified.

**Proteins name**
Tyrosine-protein kinase Lck
Secretory carrier-associated membrane protein 3
Erythrocyte band 7 integral membrane protein
Sarcoplasmic/endoplasmic reticulum calcium ATPase 2
Solute carrier family 2, facilitated glucose transporter member 1
60 kDa heat shock protein, mitochondrial
Prohibitin-2
Keratin, type II cytoskeletal 5
Dolichyl-diphosphooligosaccharide--protein glycosyltransferase subunit 1
Ig gamma-1 chain C region
Leukosialin
Tubulin alpha-1B chain
Keratin, type I cytoskeletal 9
Keratin, type II cytoskeletal 2 epidermal
Dipeptidyl peptidase 4
Isoform CD6B of T-cell differentiation antigen CD6 OS=Homo sapiens GN=CD6
B-cell receptor-associated protein 31
Prohibitin
Sodium/potassium-transporting ATPase subunit alpha-1
Solute carrier family 2, facilitated glucose transporter member 3
Myosin-9
CD44 antigen
Keratin, type I cytoskeletal 10
Tubulin beta chain
Protein disulfide-isomerase A3
Antigen peptide transporter 1
Keratin, type II cytoskeletal 1
Actin, cytoplasmic 1
Transferrin receptor protein 1
P-selectin glycoprotein ligand 1
Isoform 2 of 4F2 cell-surface antigen heavy chain
HLA class I histocompatibility antigen, Cw-12 alpha chain

Table S2: Human T-cells were activated for 8 h with anti-CD3/CD28 antibodies in the presence of recombinant human IL-2 and 10 µg/ml of Brefeldin A. Subsequently, the cells were fixed and permeabilized and prepared for intracellular staining. Antibodies to IFN-γ, IL-4 and IL-17A were used to identify the Th1 subset, the Th2 subset and Th17 subsets respectively. Data are derived from the gated $CD4^+$ cell population.

	Type-1 T helper (Th1)	Type-2 T helper (Th2)	Type-0 T helper (Th0)	Type-17 T helper (Th17)	Th17/Th1-like	Not stained
**T-cells healthy donor 1**	7.20%	30.40%	9.20%	10.80%	8.40%	34.00%
**T-cells healthy donor 2**	5%	63.70%	10.90%	0.20%	0.00%	20.20%
**T-cells healthy donor 3**	0.00%	27.70%	0.54%	29.30%	0.20%	42.26%
**T-cells healthy donor 4**	1.59%	30.00%	1.75%	0.27%	0.00%	66.56%
**T-cells healthy donor 5**	4.91%	48.40%	3.37%	0.27%	0.00%	43.05%
**T-cells healthy donor 6**	0.55%	34.50%	2.52%	0.21%	0.00%	62.22%
**T-cells Psoriasis donor 1**	1.94%	5.58%	1%	3.60%	4.70%	83.18%
**T-cells Psoriasis donor 2**	0.54%	4.34%	0.00%	0.15%	0.33%	94.64%
**T-cells Psoriasis donor 3**	0.14%	46.40%	0.07%	0.50%	0.00%	52.89%

REFERENCES

Abusamra, D.B., Al-Kilani, A., Hamdan, S.M., Sakashita, K., Gadhoum, S.Z., and Merzaban, J.S. (2015). Quantitative Characterization of E-selectin Interaction with Native CD44 and P-selectin Glycoprotein Ligand-1 (PSGL-1) Using a Real Time Immunoprecipitation-based Binding Assay. *J Biol Chem* 290**,** 21213-21230.

Acestor, N., Panigrahi, A.K., Ogata, Y., Anupama, A., and Stuart, K.D. (2009). Protein composition of Trypanosoma brucei mitochondrial membranes. *Proteomics* 9**,** 5497-5508.

Shirure, V.S., Liu, T., Delgadillo, L.F., Cuckler, C.M., Tees, D.F., Benencia, F., Goetz, D.J., and Burdick, M.M. (2015). CD44 variant isoforms expressed by breast cancer cells are functional E-selectin ligands under flow conditions. *Am J Physiol Cell Physiol* 308**,** C68-78.
